# *Toxoplasma gondii* in Egyptian Blood Donors and Livestock: Seroprevalence, Risk Factors, and One Health Implications

**DOI:** 10.3390/microorganisms14081627

**Published:** 2026-07-26

**Authors:** Marwa A. Gouda, Sahar M. Selim, Eman Fathi Fadel, Eman Attia Elmorsy, Aya Abdallah Seleem, Hany M. Ibrahim, Reda Abdel Latif Ibrahem, Yomna Mohamed Hegazy, Kholoud Adel Alsawy, Mustafa Adel A. Younis, Khaled A. Abd El-Razik, Ehab Ali Fouad, Eman L. Shehata, Tameem M. A. Mohammed, Shrouq Moustafa Tolba, Ann Hegazy, Mona Wagdy Ayad, Shaimaa A. Farag

**Affiliations:** 1 Clinical and Molecular Parasitology Department, National Liver Institute, Menoufia University, Shebin El-Kom 32511, Egypt; marwa_goda@liver.menofia.edu.eg (M.A.G.); sahar.selim@liver.menofia.edu.eg (S.M.S.); youmnahegazy20@gmail.com (Y.M.H.); 2 Medical Parasitology Department, Faculty of Medicine, Sohag University, Sohag 82524, Egypt; emanfathy@med.sohag.edu.eg; 3 Medical Parasitology Department, Faculty of Medicine, Alexandria University, Alexandria 21526, Egypt; eman.elmorsy@alexmed.edu.eg; 4 Zoonoses Department, Faculty of Veterinary Medicine, Cairo University, Giza 12613, Egypt; ayaseleem9@gmail.com; 5 Zoology Department, Faculty of Science, Menoufia University, Shebin El-Kom 32511, Egypt; hany.mohamed@science.menofia.edu.eg; 6 Public Health and Community Medicine Department, Faculty of Medicine, Menoufia University, Shebin El-Kom 32511, Egypt; reda.ibrahim@med.menofia.edu.eg (R.A.L.I.); kholoud.adel3170@med.menofia.edu.eg (K.A.A.); 7 Clinical Pathology Department, Faculty of Medicine, Sohag University, Sohag 82524, Egypt; mustatayounis1982@gmail.com; 8 Department of Animal Reproduction, Veterinary Research Institute, National Research Centre, Dokki 12622, Egypt; khaled707@hotmail.com (K.A.A.E.-R.); 9 Department of Zoonosis, Veterinary Research Institute, National Research Centre, Dokki 12622, Egypt; ehabfoaud@gmail.com (E.A.F.); 10 Clinical Pathology Department, National Liver Institute, Menoufia University, Shebin El-Kom 32511, Egypt; emanlabib2009@gmail.com (E.L.S.); tameem.mohamed@liver.menofia.edu.eg (T.M.A.M.); 11 Department of Clinical Pathology, Faculty of Medicine, Suez Canal University, Ismailia 41522, Egypt; sh.m.tolba@med.suez.edu.eg (S.M.T.); ann_hegazy@med.suez.edu.eg (A.H.); 12 Clinical Pathology Department, Faculty of Medicine, Alexandria University, Alexandria 21526, Egypt; monawagdy16@yahoo.com

**Keywords:** *Toxoplasma gondii*, toxoplasmosis, transfusion safety, seroprevalence, blood donors, One Health, zoonotic infection, Egypt

## Abstract

*Toxoplasma gondii* is a globally prevalent zoonotic parasite with major public health and veterinary impacts. In Egypt, One Health data on its distribution among blood donors and livestock are limited. This multicentric cross-sectional study aimed to determine the seroprevalence of *Toxoplasma gondii* among asymptomatic blood donors and livestock across six different Egyptian governorates and to identify the associated risk factors. Serum samples of 2000 asymptomatic blood donors and 500 animals (cattle and buffaloes) were tested for anti-*Toxoplasma gondii* IgG and IgM antibodies using ELISA. Sociodemographic and behavioral data were collected using a structured questionnaire. Overall IgG seroprevalence in blood donors was 41.8%, with IgM seropositivity at 2.1%. Significant regional variation was observed (*p* < 0.001), ranging from 66.5% in Alexandria to 16% in Cairo. The most significant risk factors were consumption of undercooked meat, contact with cats, and engagement in agricultural activities (*p* < 0.001). Among livestock, IgG and IgM seroprevalence were 7.2% and 5.2%, respectively, indicating active parasite circulation. The presence of seropositivity among apparently healthy donors raises concerns regarding transfusion safety. These findings highlight substantial and regionally heterogeneous *Toxoplasma gondii* exposure in Egypt. A One Health approach integrating public health education, food safety measures, and targeted screening of high-risk populations and animals is essential to reduce disease burden.

## 1. Introduction

The One Health framework recognizes the intrinsic interconnection between human, animal, and environmental health and requires collaborative action to manage complex zoonotic risks. This framework is actively supported by international organizations, such as the World Health Organization (WHO), the Food and Agriculture Organization (FAO), and the World Organization for Animal Health (OIE), which collaborate to implement global health initiatives. The success of this approach in controlling several zoonotic epidemics highlights its relevance to diseases with multi-host transmission dynamics. *Toxoplasma gondii* (*T. gondii*), an obligate intracellular protozoan, causes toxoplasmosis, a zoonotic disease found worldwide across terrestrial and marine environments, making it an ideal model for the application of the One Health approach [1].

The host range of *T. gondii* is exceptionally broad, encompassing nearly all warm-blooded animals, including humans, livestock, and avian species. It remains a major public health and veterinary concern because of its association with congenital infections and detrimental effects on animal productivity [2]. It is estimated that over one billion people worldwide are infected with *T. gondii*. Felids, particularly domestic cats, serve as definitive hosts responsible for environmental contamination, whereas humans and other animals function as intermediate hosts, sustaining transmission cycles [3]. In Egypt, domestic and stray cats are ubiquitous in both urban and rural settings, contributing to widespread environmental contamination with *T. gondii* oocysts and facilitating transmission to humans and livestock [4,5].

Human toxoplasmosis is mainly acquired through the oral intake of either tissue cysts in inadequately cooked meat or oocysts contaminating food, water, and soil. Ingestion of contaminated milk is a less common but notable route of infection. Less commonly, transmission may occur via organ transplantation or blood transfusion from infected donors [6]. Globally, seroprevalence estimates vary widely, ranging from 10% to 80%, with marked variations across continents [7]. A high prevalence of toxoplasmosis has been reported in Africa, Southeast Asia, the Middle East, Central/Eastern Europe, and Latin America. Variable prevalence rates have been reported in Asia (13.3−85.3%), Europe (40−76%), Africa (21.74−74.8%), and North America (7.3−26.5%) [8,9], reflecting differences in environmental conditions, dietary habits, and socio-cultural practices [7]. Although infection is typically asymptomatic in immunocompetent individuals, it can lead to severe and potentially fatal outcomes in immunocompromised patients and developing fetuses [10].

Blood transfusion is an essential medical intervention, with approximately 92 million donations collected annually worldwide; however, approximately 1.6 million units are discarded due to infectious risks [11]. Current screening protocols primarily target viral and bacterial pathogens, including the human immunodeficiency virus (HIV), hepatitis B and C viruses, and *Treponema pallidum*. However, increasing evidence underscores the need to consider *T. gondii* as a potential transfusion-transmitted infection (TTI) [12]. Notably, tachyzoites can survive in refrigerated blood for prolonged periods, raising concerns about transmission through stored blood products, particularly those rich in leukocytes [13]. Given the high prevalence of asymptomatic infections, the absence of routine screening for toxoplasmosis may represent an underrecognized risk factor for transfusion safety [14,15].

From a veterinary and economic perspective, toxoplasmosis is a major cause of reproductive failure in livestock, resulting in substantial financial loss. Ruminants, in particular, play a key role in human infection through the consumption of undercooked meat or contaminated milk [5,16]. Globally, the disease is responsible for abortions, stillbirths, and neonatal mortality, with estimated annual losses in the sheep and goat industries reaching millions of dollars [17,18]. In cattle and buffaloes, although clinical disease is less frequent, subclinical infection can still affect productivity and serve as a potential source of human exposure via the same foodborne routes [19].

Despite numerous studies in Egypt addressing *T. gondii* seroprevalence and associated risk factors, the existing evidence remains fragmented, with marked regional variability and limited integration of human and animal data [2]. Furthermore, studies specifically targeting blood donors, a critical population for transfusion safety, are scarce [20,21,22].

Previous studies investigating the seroprevalence of *T. gondii* in Egypt have primarily focused on single governorates or single-center designs, limiting their ability to capture regional variability and broader transmission patterns in Egypt. Moreover, these studies rarely incorporated an integrated One Health approach that considers both human and animal populations. To address these gaps, the present study adopted a multicentric design encompassing multiple Egyptian governorates and included both human blood donors and livestock. This comprehensive approach provides a broader epidemiological perspective by enabling the assessment of regional heterogeneity and integrated human–livestock transmission patterns within a One Health framework. While individual studies have investigated *T. gondii* in specific livestock species or single regions, to the best of our knowledge, no previous Egyptian study has simultaneously investigated both human and animal populations across multiple governorates using a unified One Health protocol. The current study was designed to provide a direct comparison of seroprevalence and risk factors across six geographically distinct regions, with the aim of generating robust evidence for region-specific control strategies and addressing the fragmentation that has characterized Egyptian toxoplasmosis research to date.

## 2. Materials and Methods

### 2.1. Ethical Considerations

This study was approved by the Institutional Review Board of the National Liver Institute, Menoufia University (NLI IRB No. 00651/2024), and the Medical Research Ethics Committee of the Faculty of Medicine, Sohag University (IRB No. SohMed24806PD). Written informed consent was obtained from all human participants after explanation of study objectives. Confidentiality was maintained through anonymized coded identifiers. Animal procedures complied with Egyptian regulations governing research and publication ethics.

### 2.2. Study Design and Setting

This multi-centric cross-sectional analytical study was conducted between August 2024 and July 2025 across six Egyptian governorates representing major geographic regions: Menoufia (Delta region, north of Cairo), Cairo (Greater Cairo, the capital), Sohag (Upper Egypt, southern Nile Valley), Alexandria (North Coast, Mediterranean Sea), Al-Sharqia, and Ismailia (Canal region, adjacent to the Suez Canal). Five centers were selected to cover these governorates.

### 2.3. Study Population and Sampling

A total of 2000 asymptomatic human blood donors and 500 animals (cattle and buffaloes) were included in the study. From each center, 400 human and 100 animal serum samples were collected. Human participants were recruited from blood donation centers using predefined inclusion and exclusion criteria for the study. The inclusion criteria were age ≥ 18 years, provision of informed consent, and negative screening for hepatitis B surface antigen (HBsAg), hepatitis C virus (HCV), and HIV. The exclusion criteria were age < 18 years and a history of chronic disease. Animal samples were obtained from cows and buffaloes owned by smallholders during the veterinary campaigns.

A multistage random sampling technique was employed: governorates were randomly selected to represent geographic regions; within each governorate, the main blood donation center was chosen by simple random sampling; and individual participants were recruited according to eligibility criteria. Animal samples were collected concurrently from the same governorates to ensure a balanced distribution.

### 2.4. Sample Size Calculation

For humans, the sample size was estimated based on a previously reported seroprevalence of 59.6% among Egyptian blood donors [20]. Assuming a 95% confidence interval and a 5% acceptable error margin, we calculated the minimum sample size as 400 individuals per center based on the standard formula:

*n* = *Z*^2^ × *p* (1 − *p*)/*e*^2^

In this equation, *n* denotes the required sample size, *Z* represents the standard normal deviate, *p* stands for the anticipated prevalence proportion, and *e* indicates the tolerable sampling error. To enhance geographic representativeness, the sample size was expanded across five centers to reach a total of 2000 participants.

For animals, the sample size was based on reported seroprevalence rates of 9.7% [23] and 7.4% [24]. Using the same formula, the minimum required sample size was approximately 470. To ensure statistical power and account for potential exclusions, 500 samples were collected from five centers.

### 2.5. Sample Collection and Processing

Approximately 3 mL of venous blood was aseptically collected from each participant. For the animals, 10 mL of blood was obtained via jugular venipuncture. Animal samples were collected from cows and buffaloes owned by smallholders during veterinary campaigns, using a convenience-based approach.

Blood samples were centrifuged at 3000 rpm for 10 min, and the sera were separated and stored at −20 °C until analysis. Available data on animal characteristics were recorded.

### 2.6. Laboratory/Serological Analysis

All serum samples were processed at the Parasitology Laboratory, Clinical and Molecular Parasitology Department, National Liver Institute, Menoufia University.

Human serum samples were screened for specific IgG and IgM antibodies against *T. gondii* using commercially available ELISA kits (PerkinElmer Health Sciences, Shelton, CT, USA; catalog numbers 10234 for IgG and 10235 for IgM). All procedures strictly adhered to the manufacturer’s protocols.

Animal sera were tested using ELISA kits (BT LAB, Shanghai, China) for the qualitative detection of anti-*T. gondii* IgG (Cat. No. ED0056Bo) and IgM (Cat. No. ED0063Bo), according to the manufacturer’s protocols.

All assays were performed in duplicate, and positive and negative controls were included in each run to ensure the reliability and reproducibility of the assay.

Molecular detection of *T. gondii* by conventional PCR was performed on a subset of samples as part of a parallel investigation; however, these data fall outside the scope of the present study and will be presented separately.

### 2.7. Data Collection

A structured questionnaire was administered to human participants to collect sociodemographic data (age, sex, residence, education, income, marital status, occupation, and crowding index). The crowding index was defined as the ratio of household members to the number of rooms, excluding kitchens and bathrooms. Following the Egyptian Demographic and Health Survey and validated socioeconomic scales for Egypt, households were classified as follows: no crowding (<1 person per room), crowding (=1 person per room), and severe crowding (>1 person per room) [25,26]. The potential risk factors assessed included dietary habits (undercooked meat and unwashed fruits/vegetables), cat contact, agricultural activities, soil exposure, hand hygiene, and water source. For animals, data on species, sex, and season were also recorded.

Participants were also provided with an informational brochure to raise awareness of toxoplasmosis, covering its transmission, clinical features, diagnosis, and prevention.

### 2.8. Statistical Analysis

Data were analyzed using SPSS version 26 (SPSS Inc., Chicago, IL, USA). Categorical variables are expressed as frequencies and percentages. The associations between variables were assessed using the Pearson chi-square (χ^2^) test. Binary logistic regression analysis was performed to identify the independent predictors of *T. gondii* IgG seropositivity. A *p* value of <0.05 was considered statistically significant.

## 3. Results

This study recruited 2000 participants. The majority (94.4%) were males, with the remaining 5.6% being females. More than half of the participants were under the age of 29 (53.3%), lived in rural areas (50.9%), were married (64.8%), had a secondary level of education (42.7%), had a sufficient income (64.4%), and had a severe crowding index (57.2%). In terms of toxoplasmosis risk factors, 28.7% of the participants consumed undercooked meat; 24.6% consumed unwashed raw vegetables and fruits; 35.4% reported being involved in gardening or agricultural practices; 55.4% reported hand washing before eating; 23.5% had contact with cats; 38.9% reported working with soil; 25.6% reported drinking untreated water; and only 12.8% had a history of blood transfusion (Table 1).

**Figure 1:**
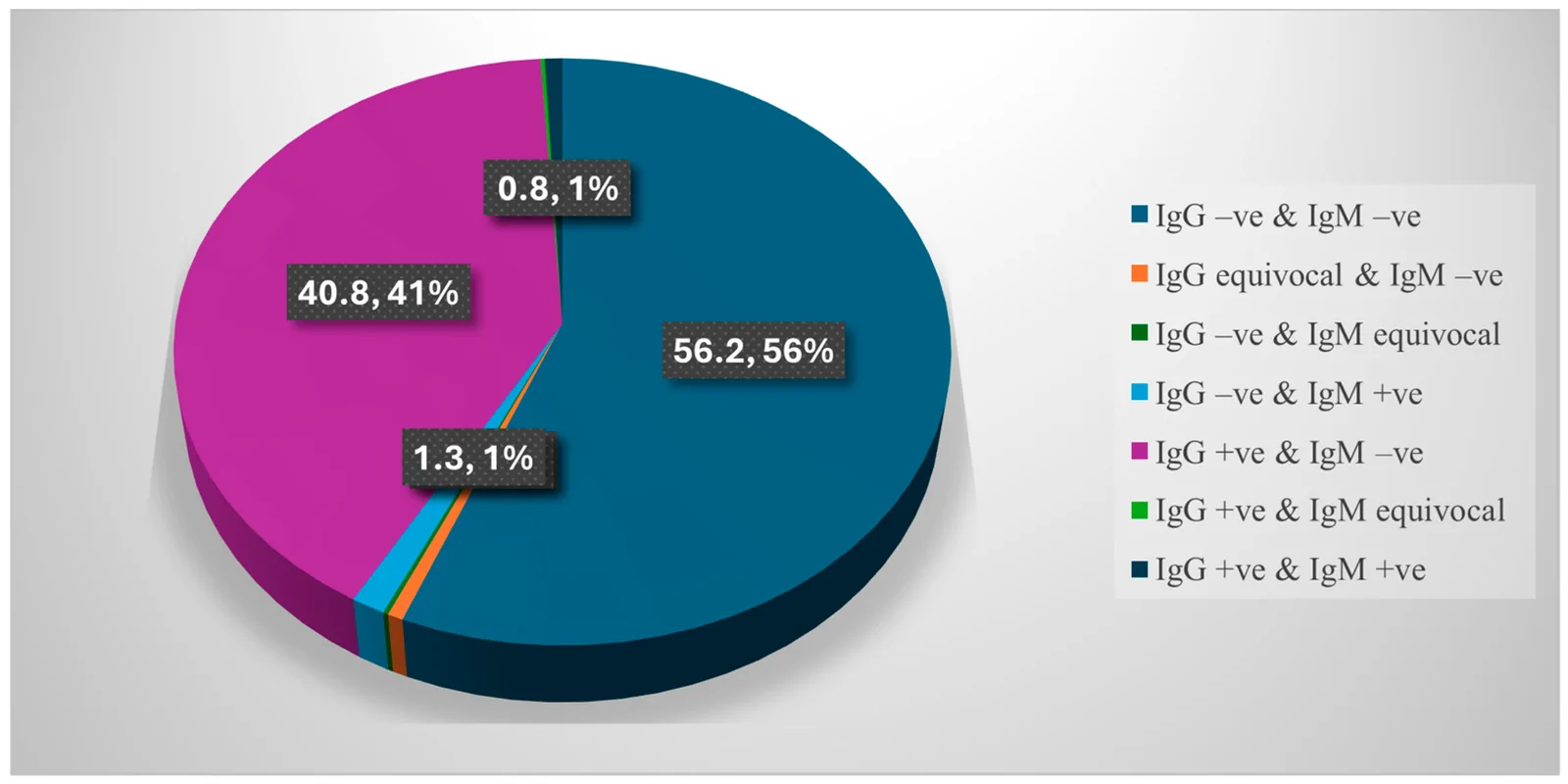
Toxoplasmosis serology (IgG and IgM) among the studied cases.

Toxoplasmosis IgM seroprevalence was positive in 2.1% of the study participants, while the seroprevalence of IgG was positive in 41.8%. Of this seroprevalence, 0.8% were positive for both IgM and IgG, 1.3% were positive for IgM and negative for IgG, and 40.8% were positive for IgG only (Table 2, Figure 1). Statistically significant geographical heterogeneity in *T. gondii* seroprevalence was observed across Egyptian governorates for both IgM and IgG (*p* < 0.001 for both). The highest IgM seroprevalence was in Cairo (6.5%), while the lowest was in Menoufia (0.3%). The highest IgG seroprevalence was in Alexandria (66.5%), while the lowest was in Cairo (16%) (Table 2).

Toxoplasmosis IgG seropositivity was statistically more prevalent among participants who consumed undercooked meat and raw vegetables and fruits; engaged in agricultural practices; had contact with cats; and dealt with soil (*p* < 0.05) (Table 3).

Binary logistic regression was performed to detect relevant risk factors for toxoplasmosis IgG seropositivity. On univariable analysis, undercooked meat, raw vegetables, agricultural activity, contact with cats, and dealing with soil were significant independent risk factors for toxoplasmosis IgG seropositivity; therefore, they were included in the multivariable analysis, which highlighted undercooked meat, agricultural activity, and contact with cats as significant independent risk factors for toxoplasmosis IgG seropositivity (*p* < 0.05) (Table 4).

The percentages for the three smallest categories, not individually labeled in the chart due to space, are: IgG equivocal & IgM negative, 0.6%; IgG negative & IgM equivocal, 0.2%; IgG positive & IgM equivocal, 0.2% (together with the four labeled categories, totaling 100%).

Regarding the seroprevalence of toxoplasmosis among the studied animals, the overall IgM seroprevalence was 5.2%, with the highest prevalence detected in the Sohag governorate (10%), while the lowest was in the Ismailia and Al-Sharqia governorates (2%). The overall seroprevalence of IgG was 7.2%, with the highest prevalence detected in the Cairo governorate, and the lowest was in the Ismailia and Al-Sharqia governorates, where none of the animals showed IgG seropositivity (0%) (Table 5).

## 4. Discussion

Toxoplasmosis is one of the most widespread zoonotic infections worldwide, posing considerable challenges to both human and animal health [2]. To our knowledge, this is one of the largest multicentric One Health studies in Egypt, integrating human and animal seroprevalence across geographically distinct regions. In this multicentric study, the overall seroprevalence of *Toxoplasma gondii* IgG antibodies among asymptomatic blood donors was 41.8%, reflecting a substantial level of prior exposure within the studied population. This relatively high prevalence suggests sustained environmental exposure and ongoing transmission cycles, reinforcing the endemicity of toxoplasmosis in Egypt.

Significant geographical heterogeneity in *T. gondii* seroprevalence was observed across Egyptian governorates, ranging from 66.5% in Alexandria to 16% in Cairo (*p* < 0.001). This statistically significant variation indicates genuine regional differences rather than sampling fluctuations. The observed patterns are likely shaped by a combination of environmental, climatic, and socioeconomic factors. For example, Alexandria’s coastal climate may enhance oocyst survival, while regional variations in diet, sanitation, and livestock contact could further influence transmission. These results highlight the focal nature of toxoplasmosis endemicity in Egypt and support region-specific rather than nationally uniform control strategies. This finding has important public health implications, as it suggests that interventions should be tailored to the epidemiological profiles of each region.

The present findings should also be interpreted in the context of previously published studies from Egypt, which have largely been limited to single-center or single governorate designs. In contrast to earlier work, including a prior study conducted in Menoufia Governorate by the same first author [21], the current study expands the epidemiological perspective through its multicentric design and inclusion of multiple geographically distinct regions. Moreover, the integration of human and animal data within a One Health framework provides additional insights into potential transmission pathways and environmental exposure, offering a more comprehensive understanding of the epidemiology of *T. gondii* in Egypt.

These findings aligned closely with earlier Egyptian studies, which reported seroprevalence rates of 65.3% among Alexandrian donors [27], 59.6% in Mansoura [20], and 45% in Fayoum [22]. In contrast, lower rates had been reported in Menoufia (~11%) [21,28], suggesting possible temporal changes in exposure. Similar variability had been documented across Arab countries, with seroprevalence estimates of 19.5% in Makkah Al Mukarramah, 33.5% in Tripoli, and 44% in Tunisia [5,29,30]. Globally, the findings of the present study fall within the intermediate range, comparable to reports from Greece (33%) [31] and Romania (45.9%) [32], but lower than those observed in northern Iran (73.5%) [33] and higher than estimates from Serbia, Taiwan, and China [3,34,35]. Collectively, these comparisons highlight the strong influence of geography, climate, and cultural practices on toxoplasmosis epidemiology.

No significant associations were observed between *T. gondii* seropositivity and sociodemographic characteristics such as age, sex, residence, marital status, educational level, income, or crowding index. This suggests that exposure to infection is widespread across the studied population rather than being confined to specific demographic groups.

These results were consistent with those of Sarkari et al. [36], who reported no association between sex and *T. gondii* seropositivity, attributing this to the predominance of male participants in blood donor populations, a profile similar to that of the current study. The predominance of male participants (94.4%) in this study reflects the demographic profile of blood donors in Egypt, where males constitute the vast majority of voluntary blood donors due to cultural and social factors. Blood donation campaigns in Egypt primarily target males, as females are often underrepresented due to cultural norms, a higher prevalence of anemia, and pregnancy-related deferrals. This gender distribution is consistent with previous studies on blood donors in Egypt and other Middle Eastern countries [20,21,28]. In contrast, Gouda et al. [21] stated that no significant associations were observed between *T. gondii* seropositivity and sociodemographic characteristics, except for sex, as 15.4% of males and 50% of females were positive, indicating that female sex was a major risk factor in this study. Moreover, Henin et al. [22] found that the prevalence of IgG seropositivity increased in those aged ≥ 30 years, and there was a significant correlation between toxoplasmosis and rural residence (86.2%). The results of the current study can be explained by the absence of age-related differences, which may reflect early-life exposure and cumulative infection over time. The lack of association with sex is likely influenced by the predominance of male participants, reflecting the demographic profile of blood donors rather than true biological differences [36]. Similarly, the non-significant associations with residence and socioeconomic indicators indicate that transmission is more strongly driven by environmental and behavioral factors than by demographic characteristics.

The prevalence of anti-*T. gondii* IgM antibodies was relatively low (2.1%), indicating that most infections in this population are likely chronic rather than recent. Nevertheless, significant regional variation was observed (*p* < 0.001), with the highest IgM prevalence in Cairo (6.5%) and the lowest in Menoufia (0.3%), suggesting possible differences in recent exposure across different settings. Comparably low IgM seroprevalence has been reported in other studies, including 0.5% and 0.41% [3,37], whereas slightly higher rates have been documented in Menoufia (5.5% and 5.9%) [21,28]. These variations may reflect differences in the study populations, environmental exposure, and diagnostic approaches. Although IgM positivity was uncommon, its presence remains clinically relevant, particularly in the context of transfusion safety, as it may indicate recent or potentially transmissible infections.

Several behavioral and environmental factors were significantly associated with seropositivity. Undercooked meat consumption was the most potent independent risk factor, reinforcing the established understanding that tissue cyst ingestion is the primary transmission route. Engagement in agricultural activities and contact with cats were also identified as independent risk factors, highlighting the importance of environmental exposure and the role of felids in environmental contamination with oocysts. Although the consumption of raw vegetables and contact with soil were significant in the univariate analysis, they did not remain independent predictors after adjustment, suggesting possible confounding or shared exposure pathways that require further investigation. These findings underscore the substantial contributions of both foodborne and environmental transmission routes to the transmission dynamics in the studied population. These results were consistent with those of Elsheikha et al. [20], Gouda et al. [21], and Ibrahem et al. [28].

From a One Health perspective, the inclusion of animal data provides important insights into the transmission dynamics of *T. gondii* infection. The observed seroprevalence among cattle and buffaloes (7.2% for IgG and 5.2% for IgM) confirmed the circulation of the parasite within livestock populations. The concordance between human and animal findings supports the existence of shared environmental sources of infection and ongoing zoonotic transmission of the disease. These results were consistent with studies from the Sohag (7%) [38], Kafr El Sheikh, Gharbia, and Qalyubia governorates in the Nile Delta of Egypt (7.4%) [24] and Qena (9.1%) [23], although lower (3%) [39] and considerably higher rates in other regions, such as Aswan and Assiut, have also been reported [5,40]. Such variability likely reflects differences in environmental conditions, animal husbandry practices, and levels of exposure to feline contamination.

Global prevalence estimates vary widely (0–87.79%) depending on the regional parasite genotypes [41]. These findings fall within this range and align with studies from China (7.5%) [42] and Iraq (7.4%) [43].

In Egypt, the endemicity of toxoplasmosis is driven by a combination of ecological and socioeconomic factors, including the widespread presence of stray and domestic cats, traditional farming systems, and close human–animal interactions [2,44,45]. While small ruminants are traditionally considered the primary reservoirs, increasing evidence suggests that cattle and buffaloes may also contribute to the transmission dynamics [2,46].

From a transfusion medicine perspective, the relatively high IgG seroprevalence among apparently healthy donors is noteworthy. Although IgG positivity reflects past infection, the potential for transient parasitemia raises concerns about transfusion-transmitted toxoplasmosis, particularly in immunocompromised recipients. The absence of routine screening for *T. gondii* in blood donation programs may represent an under-recognized gap in blood safety protocols [12].

Despite its strengths, including the large sample size and multicentric design, this study has some limitations. The cross-sectional design limited the ability to establish causal relationships between identified risk factors and *T. gondii* infection. In addition, the use of serological assays does not allow precise differentiation between recent and chronic infections. Although molecular analysis was performed on a subset of samples, the present study focused on serological findings; integration of molecular data will be addressed in future investigations. Furthermore, the study population, comprising blood donors and animals from selected sites, may not fully represent the general population, which could limit the generalizability of these findings. Finally, potential clustering effects across the study locations were not explicitly accounted for, which may have influenced regional comparisons.

## 5. Conclusions

In summary, this study provides robust epidemiological evidence of active *T. gondii* circulation in Egypt and underscores the critical value of a One Health strategy. The high seroprevalence among asymptomatic blood donors reflects widespread exposure, with undercooked meat consumption, agricultural activities, and contact with cats identified as key drivers of infection, emphasizing both foodborne and environmental transmission pathways. The detection of infection in livestock further supports the shared human–animal transmission cycle. These findings underscore the need for integrated control strategies, including public health education, food safety measures, veterinary surveillance, and targeted screening of high-risk groups, to enhance transfusion safety. Future studies incorporating molecular approaches and longitudinal designs are warranted to better understand transmission dynamics and inform effective prevention strategies.

## Figures and Tables

**Table 1:** Sociodemographic profile and risk factors of the study participants (N = 2000).

Variable		N (%)
Age (Years)	Mean ± SD	30.32 ± 9.40
	Range	18–66
Age (Years)	≤29 years	1065 (53.3)
	>29 years	935 (46.8)
Sex	Male	1887 (94.4)
	Female	113 (5.6)
Residence	Rural	1019 (50.9)
	Urban	981 (49.1)
Marital status	Single	704 (35.2)
	Married	1296 (64.8)
Educational level	Illiterate	169 (8.5)
	Basic	390 (19.5)
	Secondary	854 (42.7)
	University	587 (29.4)
Income	Less than sufficient	546 (27.3)
	Sufficient	1287 (64.4)
	More than sufficient	167 (8.4)
Crowding index	<1 (no crowding)	217 (10.9)
	=1 (crowding)	640 (32.0)
	>1 (severe crowding)	1143 (57.2)
Consumption of undercooked meat		574 (28.7)
Consumption of unwashed raw vegetables & fruits		492 (24.6)
Gardening or practice of agriculture		708 (35.4)
Hand washing before eating		1108 (55.4)
Contact with cats		469 (23.5)
Dealing with soil		778 (38.9)
Drinking untreated water		511 (25.6)
History of blood transfusion		256 (12.8)

**Table 2:** Seroprevalence of toxoplasmosis in the different Egyptian governorates studied.

Variable	Alex (N = 400)	Cairo (N = 400)	Ismailia and Al-Sharqia (N = 400)	Menoufia (N = 400)	Sohag (N = 400)	Total (N = 2000)
	N (%)	N (%)	N (%)	N (%)	N (%)	N (%)
Toxoplasmosis IgM						
Positive	9 (2.3)	26 (6.5)	2 (0.5)	1 (0.3)	3 (0.8)	41 (2.1)
Equivocal	4 (1.0)	0 (0.0)	1 (0.3)	0 (0.0)	2 (0.5)	7 (0.4)
Negative	387 (96.8)	374 (93.5)	397 (99.3)	399 (99.8)	395 (98.8)	1952 (97.6)
χ^2^ (*p* value)	62.10 (<0.001) *					
Toxoplasmosis IgG						
Positive	266 (66.5)	64 (16.0)	192 (48.0)	150 (37.5)	163 (40.8)	835 (41.8)
Equivocal	9 (2.3)	3 (0.8)	0 (0.0)	0 (0.0)	0 (0.0)	12 (0.6)
Negative	125 (31.3)	333 (83.3)	208 (52.0)	250 (62.5)	237 (59.8)	1153 (57.7)
χ^2^ (*p* value)	251.14 (<0.001) *					

* Statistically significant; χ^2^: chi-squared test.

**Table 3:** Relationship between toxoplasmosis IgG seropositivity and sociodemographic, clinical data, and risk factors among the participants (N = 2000).

Variable	Toxoplasmosis IgG		χ^2^	*p* Value
	Negative and Equivocal (N = 1165)	Positive (N = 835)		
	N (%)	N (%)		
Sex				
Male	1108 (95.1)	779 (93.3)	3.00	0.083
Female	57 (4.9)	56 (6.7)		
Age (Years)				
≤29 years	618 (53.0)	447 (53.5)	0.05	0.830
>29 years	547 (47.0)	388 (46.5)		
Residence				
Rural	614 (52.7)	405 (48.5)	3.44	0.064
Urban	551 (47.3)	430 (51.5)		
Marital status				
Single	412 (35.4)	292 (35.0)	0.03	0.855
Married	753 (64.6)	543 (65.0)		
Educational level				
Illiterate	94 (8.1)	75 (9.0)	4.57	0.207
Basic	240 (20.6)	150 (18.0)		
Secondary	479 (41.1)	375 (44.9)		
University	352 (30.2)	235 (28.1)		
Income				
Less than sufficient	302 (25.9)	244 (29.2)	3.14	0.208
Sufficient	768 (65.9)	519 (62.2)		
More than sufficient	95 (8.2)	72 (8.6)		
Crowding index				
<1 (no crowding)	126 (10.8)	91 (10.9)	0.37	0.831
=1 (crowding)	379 (32.5)	261 (31.3)		
>1 (severe crowding)	660 (56.7)	483 (57.8)		
Undercooked meat	237 (20.3)	337 (40.4)	95.23	<0.001 *
Raw vegetables & fruits	267 (22.9)	225 (26.9)	4.25	0.039 *
Agricultural activity	342 (29.4)	366 (43.8)	44.57	<0.001 *
Hand washing	628 (53.9)	480 (57.5)	2.52	0.112
Contact with cats	227 (19.5)	242 (29.0)	24.44	<0.001 *
Dealing with soil	418 (35.9)	360 (43.1)	10.71	0.001 *
Untreated water	300 (25.8)	211 (25.3)	0.06	0.808
Blood transfusion	136 (11.7)	120 (14.4)	3.17	0.075

* Statistically significant; χ^2^: chi-squared test. Bold values indicate statistical significance (*p* < 0.05).

**Table 4:** Logistic regression for relevant risk factors for toxoplasmosis IgG seropositivity.

Variable	Univariable				Multivariable			
	OR	95% CI		*p* Value	OR	95% CI		*p* Value
		Lower	Upper			Lower	Upper	
Undercooked meat								
Yes	2.65	2.17	3.23	<0.001 *	2.49	2.02	3.06	<0.001 *
No	1.0				1.0			
Raw vegetables & fruits								
Yes	1.24	1.01	1.52	0.039 *	0.88	0.70	1.10	0.262
No	1.0				1.0			
Agricultural activity								
Yes	1.88	1.56	2.26	<0.001 *	1.74	1.41	2.14	<0.001 *
No	1.0				1.0			
Contact with cats								
Yes	1.69	1.37	2.08	<0.001 *	1.53	1.23	1.90	<0.001 *
No	1.0				1.0			
Dealing with soil								
Yes	1.35	1.13	1.63	0.001 *	1.01	0.82	1.24	0.933
No	1.0				1.0			

* Statistically significant; OR: odds ratio; CI: confidence interval. Bold values indicate statistical significance (*p* < 0.05).

**Table 5:** Characteristics and toxoplasmosis seroprevalence of the studied animals.

Variable	Alex (N = 100)	Cairo (N = 100)	Ismailia and Al-Sharqia (N = 100)	Menoufia (N = 100)	Sohag (N = 100)
	N (%)	N (%)	N (%)	N (%)	N (%)
Animal type					
Buff	0 (0.0)	100 (100.0)	0 (0.0)	22 (22.0)	100 (100.0)
Cow	100 (100.0)	0 (0.0)	100 (100.0)	78 (78.0)	0 (0.0)
Animal sex					
Male	16 (16)	42 (42.0)	0 (0.0)	0 (0.0)	97 (97.0)
Female	84 (84)	58 (58.0)	100 (100.0)	100 (100.0)	3 (3.0)
Season					
Winter	0 (0.0)	43 (43.0)	0 (0.0)	22 (22.0)	0 (0.0)
Summer	100 (100.0)	57 (57.0)	100 (100.0)	78 (78.0)	100 (100.0)
IgM					
Positive	3 (3.0)	7 (7.0)	2 (2.0)	4 (4.0)	10 (10.0)
Negative	97 (97.0)	93 (93.0)	98 (98.0)	96 (96.0)	90 (90.0)
IgG					
Positive	2 (2.0)	13 (13.0)	0 (0.0)	11 (11.0)	10 (10.0)
Negative	98 (98.0)	87 (87.0)	100 (100.0)	89 (89.0)	90 (90.0)

## Data Availability

The data supporting the findings of this study are contained within the manuscript. The raw data are available from the corresponding author upon request.

## Abbreviations

The following abbreviations are used in this manuscript:

WHO	World Health Organization
OIE	World Organization for Animal Health
FAO	Food and Agriculture Organization
*T. gondii*	Toxoplasma gondii
TTI	Transfusion-transmitted infection
HBsAg	Hepatitis B surface antigen
HCV	Hepatitis C virus
HIV	Human immunodeficiency virus
ELISA	Enzyme-linked immunosorbent assay
IgG	Immunoglobulin G
IgM	Immunoglobulin M
